# Crystal Structure of Nd_10.67_Pt_4_O_24,_ a New Neodymium Platinate

**DOI:** 10.1021/acsomega.5c00031

**Published:** 2025-03-19

**Authors:** Øystein Slagtern Fjellvåg, Helmer Fjellvåg, Julie Hessevik, Anja Olafsen Sjåstad, Gwladys Steciuk

**Affiliations:** † Department for Hydrogen Technology, Institute for Energy Technology P.O. Box 40, Kjeller NO-2027, Norway; ‡ Center for Materials Science and Nanotechnology, Department of Chemistry, University of Oslo, Oslo N-0315, Norway; § Institute of Physics of the CAS, Na Slovance 1999/2, Prague 8 182221, Czech Republic

## Abstract

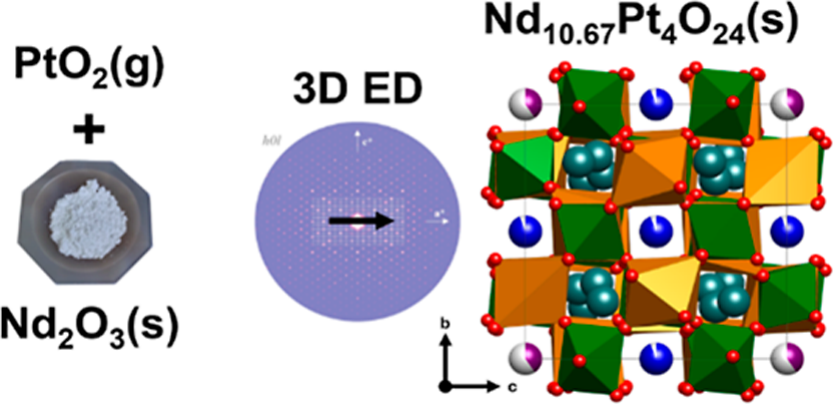

A new platinate was recently discovered when Nd_2_O_3_ was explored as a platinum capture material in the Ostwald process, formed by a direct reaction between gaseous PtO_2_ and Nd_2_O_3_. The crystal structure of this new platinate and its composition, Nd_10.67_Pt_4_O_24_, are here reported for the first time. The compound is synthesized either by a direct reaction between PtO_2_(*g*) and Nd_2_O_3_ or by the citric acid chemical route. Based on 3-dimensional electron diffraction data and Rietveld refinement of high-resolution synchrotron and neutron powder diffraction data, we describe its crystal structure in space group I4_1_/*a*. The compound is structurally related to the Ln_11–*x*_Sr_*x*_Ir_4_O_24_ (Ln = La, Pr, Nd, and Sm) phases with a double perovskite (A_2_BB’O_6_)-like crystal structure with A-site cation deficiency. Owing to the fixed oxidation state of Pt(IV), two of the four Nd sites are partly occupied to provide charge neutrality, with Nd4 taking a split position. On heating, Nd_10.67_Pt_4_O_24_ decomposes into Nd_2_O_3_ and Pt. A plateau in the thermogravimetric curves measured in 33 vol % O_2_ in N_2_ indicates the presence of an intermediate Pt(II) phase at around 960 °C, probably isostructural with La_4_PtO_7_.

## Introduction

The rare earth (Ln) cations are well-known to form pyrochlore (A_2_B_2_O_7_) type compounds together with tetravalent platinum, Ln_2_Pt_2_O_7_.^1−4^ Such pyrochlores have attracted significant attention during the last decades owing to the interesting physics of geometrically frustrated spin systems.^1,5^ The stability of pyrochlores with trivalent A and tetravalent B cations is governed by the relative size of the A and B atoms. The stability window of 0.58 ≤ *R*_B_/*R*_A_ ≤ 0.74 can be extended when turning to high-pressure synthesis routes.^1^ Nd_2_Pt_2_O_7_^3,6^ concurs with the A_2_B_2_O_7_ stability criterion^1^ but is only reported as a high-pressure compound obtained at P = 40 kbar and *T* = 1620 K. The La analogue falls outside the stability range and is not known. On the other hand, several detailed studies have been carried out for the heavier Ln platinates like Gd_2_Pt_2_O_7_.^7^

Although many rare earth platinates are conveniently synthesized by high-pressure methods, several platinates are already easily obtained by traditional solid-state reactions,^8^ e.g., double perovskites (A_2_BB’O_6_) with rare earth cations, Ln_2_BPtO_6_ (Ln = La, Pr, Nd, Sm, Eu, and Gd; B = Mg, Co, Ni, and Zn),^9^ or by growth in high-temperature carbonate fluxes, e.g., La_3_NaPtO_7_, Nd_3_NaPtO_7_, and La_4_PtO_7_.^10^ The direct reaction between the solid binary components, Ln_2_O_3_ and PtO_2_, is hampered by the low thermal stability of PtO_2_, being limited to around 600 °C.^8^ However, at high temperatures, PtO_2_ is reactive in its molecular form, and ternary platinates can be formed within an appropriate synthesis environment.^11^ Gas streams containing PtO_2_ can be achieved by passing air or oxygen over a heated Pt filament. In this way, volatile platinum species are transported in the vapor phase and can react with oxides in their solid state to form platinates; see example eqs (1) and (2).^11,12^
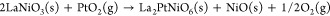
1
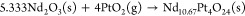
2

An extensive study described the solid-state synthesis of Ln–Pt–O compounds using mechanic mixtures of Ln_2_O_3_, PtO_2_(*s*), and Pt metal wrapped in Au foil and heat treated in sealed evacuated silica glass vials at 900 °C. In this way, Ln_2_PtO_4_ (Ln = La, Pr, Nd, Sm, Eu, and Gd) were achieved, both notably with Pt(II) cations.^8^ High-temperature crystal growth in carbonate fluxes gave interestingly both Pt(II) compounds like La_4_PtO_7_ but also Pt(IV) compounds like La_3_NaPtO_7_ and Nd_3_NaPtO_7_.^10^ The different oxidation states for platinum have a distinct structural chemistry impact with respect to local coordination. The d^8^ Pt(II) cations in La_4_PtO_7_ exhibit square planar coordination (Pt–O; 2 × 2.053 Å; 2 × 2.045 Å) with [PtO_3_]^4–^ zigzag chains of corner-sharing square planar units along the *b* axis. Similar structural features are found for a number of Pd(II) compounds, like La_4_PdO_7_.^10^ On the other hand, for Pt(IV) in La_3_NaPtO_7_, the d^6^ Pt(IV) cations take regular octahedral coordination (Pt–O; 6 × 2.023 Å).^10^

The basis for the current work is our observation that volatile PtO_2_ reacts with Nd_2_O_3_ at high temperatures and forms an unknown compound. This was noticed during our efforts on capturing volatile Pt species in high-temperature processes like the ammonia oxidation reaction. During these experiments, PtO_2_(*g*) was produced by passing air over heated Pt wires and transported in a quartz tube system before reacting with Nd_2_O_3_ pellets.^11^ This new phase was thereafter synthesized by means of the citric acid wet-chemical route, which after optimization of the cationic composition yielded a phase pure product of Nd_10.67_Pt_4_O_24_. We currently report on the crystal structure of the nonstoichiometric Nd_10.67_Pt_4_O_24_ compound, exhibiting a crystal structure similar to that of Ln_9_Sr_2_Ir_4_O_24_.^13^ Several isostructural compounds are known, including A_11_Re_4_O_24_ (A = Ca and Sr)^14,15^ and Ba_11_Os_4_O_24_.^16^ The crystal structure was solved and refined on the basis of 3-dimensional electron diffraction (3D ED),^17,18^ refined based on synchrotron powder diffraction data, and carefully evaluated with respect to neodymium nonstoichiometry and oxygen coordination by means of high-resolution powder neutron diffraction data. The oxidation state of platinum was evaluated based on thermogravimetric data for complete decomposition into Nd_2_O_3_ and Pt. This decomposition was furthermore carefully investigated by means of thermogravimetric analysis (TGA) and powder X-ray diffraction. The results are discussed in relation to the crystal structure of Ln_9_Sr_2_Ir_4_O_24_ and related compounds, as well as to other Ln platinates.

## Experimental Section

Nd_10.67_Pt_4_O_24_ was synthesized using the wet-chemical citric acid complexation method. Starting materials were Nd_2_O_3_ (99.9%, Sigma-Aldrich), Pt metal (99.9%, K. A. Rasmussen), C_6_H_8_O_7_ × H_2_O (citric acid monohydrate, purity ≥99.5%, Sigma-Aldrich), O_2_(*g*) (99.999%, AGA), HNO_3_ (65 wt %, Merck KGaA), and HCl (37%, Fischer Scientific). Prior to synthesis, Nd_2_O_3_ was annealed at 900 °C to remove any hydroxide and carbonate and then cooled in a desiccator. Nd_2_O_3_ was thereafter dissolved in 6 M HNO_3_ and Pt metal was dissolved in aqua regia (HNO_3_:HCl = 1:3). The precursor solutions were mixed to synthesize a first batch with a nominal 3:1 stoichiometric ratio between Nd and Pt. Based on preliminary phase content analysis, a second batch was made with the exact composition of the target phase, Nd_10.67_Pt_4_O_24_. Around 50 g citric acid was added to the solution per gram of Nd_10.67_Pt_4_O_24_ product. The solution was heated during boiling with the release of water and nitrous gases, followed by overnight heat treatment at 180 °C. Calcination was done at 400 °C in static air for 12 h in a muffle furnace. The finely crushed powder was pressed into cylindrical pellets using a static pressure of 100 bar. Sintering was performed in a flow of 1 atm O_2_(*g*) at 800 °C. The process was repeated twice with duration times of 24 and 72 h, respectively, with intermediate crushing and repelletizing.

**Figure 1:**
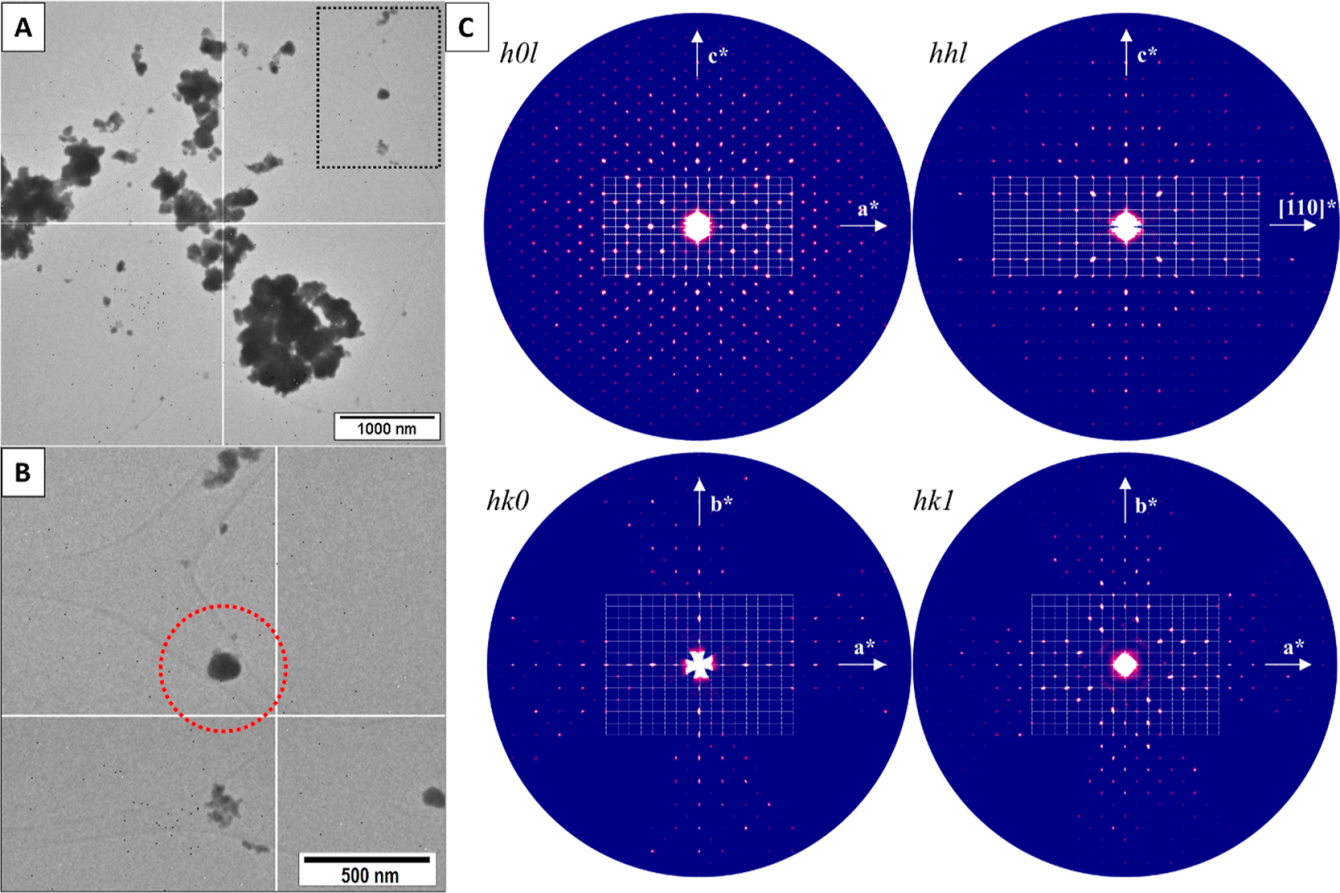
(A) Overview of the sample’s morphology under TEM. (B) Crystal selected for 3D ED analysis with the nanobeam size of about 500 nm. (C) Essential sections of the reciprocal space to define the symmetry of a tetragonal system.

For single-crystal characterization by electron diffraction, the powder was dispersed in ethanol and deposited on a Cu grid coated by a holey amorphous carbon film. 3D ED data were collected using a continuous mode (cRED) in a FEI Tecnai G2 20 TEM (acceleration voltage of 200 kV, LaB_6_) equipped with a side-mounted hybrid single-electron detector ASI Cheetah M3, 512 × 512 pixels with high sensitivity and fast readout.^17,18^ The data collection is automated by our in-house software RATS during which a series of nonoriented patterns are continuously collected by steps of 0.4° on the accessible tilt range. A dozen data sets were collected on different crystals to get an overview of the sample (Figure 1). The best data with the lowest RC width = 0.001 Å^–1^ and apparent mosaicity = 0.0473° was selected for structure characterization (Figures 1 and S1). 3D ED data reduction was performed with the program PETS2.^19−21,30^ The specific data processing for cRED data is extensively detailed in Klar et al.^21^ To model experimental intensities from continuous rotation data, Overlapping Virtual Frames (OVFs) are produced by summing consecutive experimental diffraction patterns into a set of virtual frames. Each OVF is characterized by its angular tilt range Δα_v_ covered by the virtual frame and the angular tilt step between two virtual frames (Table S1). The data reduction results in two *hkl* types of files: one assuming the kinematical approximation later used in the structural solution (and the kinematical refinement) with *R*(int)/w*R*(int) = 0.3593/0.3607 and 92% coverage for sinθ/λ = 0.75 Å^–1^ for the Laue class 4*/m* and the other one dedicated to the dynamical refinement where each OVF is independently refined.^22,23^ The structure was solved using Superflip^24,25^ in Jana2020^26^ and refined using the dynamical theory with DYNGO in Jana2020. The data collection and refinement details are presented in the Supporting Information.

Powder synchrotron X-ray diffraction data was measured at the Swiss-Norwegian Beamlines (SNBL; BM31) at the European Synchrotron Radiation Facility, Grenoble, using a wavelength of 0.25509 Å (0.3 mm glass capillary; transmission mode; LaB_6_ calibration). The data was integrated using the Bubble software into 5000 data points with a step size of 0.004. The Rietveld analysis used the structure model as obtained by 3D ED as a starting point and was carried out using the JANA^26^ suite of programs. Standard characterization in the home laboratory was done using a Bruker D8 Discover in reflection mode (CuKα_1_; Lynxeye detector) and in transmission (MoKα, 2D Eiger detector) geometry.

Powder neutron diffraction time-of-flight (TOF) data were collected on a 2.5 g powder sample at the GEM instrument^27^ at ISIS pulsed neutron and muon source, UK. The sample was mounted in a vanadium can and data was collected at room temperature. Instrumental parameters were obtained by refinement of a NIST Si standard. The diffraction data was analyzed using the TOPAS software.^28^ In the final refinement, we refined 2 lattice parameters, 26 atomic coordinates, 5 isotropic displacement parameters (individual for Pt, one common for neodymium, and one common for oxygen), and 2 occupancies (Nd3 and Nd4). In addition, instrumental parameters for each detector bank were refined.

TGA was carried out by measuring data on a NETZSCH STA-449 F1 Jupiter unit using alumina crucibles. Experiments were carried out between room temperature and 1100 °C in a 33 vol % O_2_ in N_2_ gas mixture or N_2_ gas over the sample and by using a total flow rate of 60 mL/min.

## Results and Discussion

### Crystal Structure and Structural Chemistry

Nd_10.67_Pt_4_O_24_ is formed when PtO_2_(*g*) reacts with Nd_2_O_3_ as described in eq 2. This reaction path occurs at elevated temperatures under the Ostwald process conditions for nitric acid/fertilizer production where gaseous PtO_2_(*g*) is lost from the Pt–Rh ammonia oxidation catalyst and in turn can be captured by means of an appropriate Nd_2_O_3_-based catchment system as described in Hessevik et al.^11^

The obtained gray-green powder of Nd_10.67_Pt_4_O_24_ was phase pure when prepared with a citric acid chemical route. However, for the nonoptimized sample with a nominal Nd:Pt ratio of 3:1, the Rietveld refinements revealed two small, unfitted peaks that corresponded neither to Nd_2_O_3_ nor Nd_2_Pt_2_O_7_.

To identify the crystal structure of Nd_10.67_Pt_4_O_24_, crystallites of the material were investigated by 3D ED. The indexing in PETS2^29^ first offered an F-centered pseudocubic unit cell of about *a*_cubic_ = 16.12(2) Å. A fine analysis of distortion parameters^19^ together with the first integration statistics showed instead a tetragonal body-centered unit cell with *a* = 11.3474(1) Å and *c* = 16.203(1) Å, Figure 1. The symmetry determination remained ambiguous from cRED as very strong dynamical effects partially hide the systematic extinctions related to the a-glide mirror (*h* = 2*n* on *hhl*, *hk*0, and *h*00) and the 4_1_ screw axis (*l* = 4*n* on 00*l*), Figure 1c. Therefore, the synchrotron powder data were used to evaluate the space groups and to refine the lattice parameters with better accuracy. Evaluation of the possible space groups shows that *I*4_1_/*a* provides the best fit, later confirmed by the symmetry test in SUPERFLIP.

The structure model was then obtained from cRED data in SUPERFLIP (Jana2020) and refined using the dynamical theory of electron diffraction. The dynamical refinement converged toward *R*(obs)/w*R*(obs) = 0.0902/0.0962, *R*(all)/w*R*(all) = 0.1045/0.1001 for 6991/9953 observed/all reflections and 158 refined parameters. The structural information from 3D ED is summarized in Table 1 and the atomic coordinates obtained are given in Table 2.

**Table 1:** 3D ED Data Collection and Structure Refinement Details^a^

refined structural formula (dyn)	Nd_10.732_Pt_4_O_24_
crystal system	tetragonal
*A*	11.35203(11) Å
*C*	16.21114(18) Å
*V*	2089.11(4) Å^3^
*Z*	4
density [g·cm^–3^]	8.6235
space group	I41/*a*
temperature	ambient
TEM	FEI Tecnai G2 20
radiation (wavelength)	electrons (0.0251 Å)
crystal dimensions (nm)	140 × 100
Δα/total α tilt (°)	0.4/100 data 2: 0.25/110
OVF: Δαv*/*step between OVF(°)	2.8/1.6 data 2: 1.75/1
resolution range (θ)	0.08–1.15
limiting Miller indices	*h*: −12→ 12, *k*: 0→18, *l*: 0 → 25
no. of independent reflections (obs/all)–kinematic	1906/2116
*R*_int_ (obs/all)–kinematic	0.3593/0.3607
redundancy	4.797
coverage for sinθ/λ = 0.8 Å^–1^	92.12%
kinematical refinement of nonhydrogen atoms	
no. of reflections (obs/all) up to sinθ/λ = 0.75 Å^–1^	1904/2114
extinction correction—Becker	G_iso_ = 0.474837
*R*, w*R* (obs); *R*, w*R* (all)	0.3137/0.3660; 0.3248/0.3690
*N* refined param.	37
formula	Nd_10.733(16)_Pt_4_O_24_
occupancies*:*Nd3 and Nd4	0.988(6), 0.379(5)
dynamical refinement	
no. of collected reflections (obs/all)	19,225/26,121
selection criteria RSg(max)	0.6
no. of filtered outliers for |Fobs–Fcalc|>10σ(Fobs)	27
thickness model	wedge
effective thicknesses	1018(5)
no. of reflections (obs/all)	6991/9953
GOF(obs)/ GOF(all)	0.0189/0.0166
*R*, w*R* (obs)	0.0902/0.0962
R, wR (all)	0.1045/0.1001
*N* all param./*N* struct. parameters	158/96
Rietveld refinement from synchrotron powder data	
no. of reflections (obs/all)	1244/1256
*R*, w*R* (obs)	0.0404/0.0677
*R*, w*R* (all)	0.0435/0.0677
no. of refined param. (structural ones)	35
Rp, wRp, GOF	0.0188, 0.0276, 0.1062
refined formula	Nd_10.796_Pt_4_O_24_
Rietveld refinement from neutron powder data	
no. of reflections (obs/all)	14,816/14,981
no. of refined param. (structural ones)	32
R exp, Rp, wRp, GOF	0.0204/0.0299/0.0210/1.0292
refined formula	Nd_10.696(1)_Pt_4_O_24_

a Details of Rietveld refinement of synchrotron powder data.

**Table 2:** Atomic Coordinates and Anisotropic Displacement Parameters for Nd_10.733(16)_Pt_4_O_24_ Based on Refinement of 3D ED data^a^

atom	*x*	*y*	*z*	Ueq/Uiso	Occ	Wyckoff
Nd1	0.72619(11)	0.70294(10)	0.50834(6)	0.0121(3)	1	16f
Nd2	0.79388(11)	0.47641(11)	0.66284(6)	0.0137(3)	1	16f
Nd3	1	0.5	0.49058(10)	0.0213(5)	0.988(6)	8e
Nd4	0.5	0.5	0.5342(2)	0.0179(12)	0.379(5)	8e
Pt1	0.75	0.5	0.375	0.0100(3)	1	8c
Pt2	1	0.75	0.625	0.0109(3)	1	8d
O1	0.9068(5)	0.3242(5)	0.7184(3)	0.0128(14)	1	16f
O2	0.6503(6)	0.6443(5)	0.3686(3)	0.0144(14)	1	16f
O3	0.8915(5)	0.6161(5)	0.5933(3)	0.0132(12)	1	16f
O4	0.8788(5)	0.6216(6)	0.3986(3)	0.0197(18)	1	16f
O5	0.5069(5)	0.7078(6)	0.5041(3)	0.0159(15)	1	16f
O6	0.6654(5)	0.8912(5)	0.4519(3)	0.0136(14)	1	16f
ADP harmonic parameters						
atom	U11	U22	U33	U12	U13	U23
Nd1	0.0163(6)	0.0081(5)	0.0119(4)	0.0018(6)	–0.0016(4)	0.0003(3)
Nd2	0.0120(5)	0.0094(5)	0.0197(5)	–0.0023(6)	–0.0035(4)	0.0013(4)
Nd3	0.0261(11)	0.0129(8)	0.0248(9)	0.0075(10)	0	0
Nd4	0.014(2)	0.010(2)	0.029(2)	–0.001(2)	0	0
Pt1	0.0082(6)	0.0108(6)	0.0111(5)	0.0011(7)	–0.0010(4)	–0.0001(4)
Pt2	0.0119(6)	0.0101(6)	0.0108(5)	–0.0017(7)	0.0000(4)	0.0001(5)
O1	0.011(3)	0.015(3)	0.012(2)	0.000(3)	–0.0045(18)	0.0007(18)
O2	0.020(3)	0.007(2)	0.016(2)	0.007(3)	0.003(2)	0.0036(18)
O3	0.006(3)	0.016(3)	0.018(2)	0.002(3)	0.0078(18)	0.0025(19)
O4	0.017(3)	0.025(4)	0.017(2)	–0.012(3)	–0.004(2)	0.007(2)
O5	0.010(2)	0.028(3)	0.010(2)	0.002(3)	0.0047(18)	–0.0007(19)
O6	0.014(3)	0.008(2)	0.019(2)	0.001(3)	0.004(2)	0.0090(19)

a Space group I4_1_*/a*. Calculated standard deviations in parentheses.

**Figure 2:**
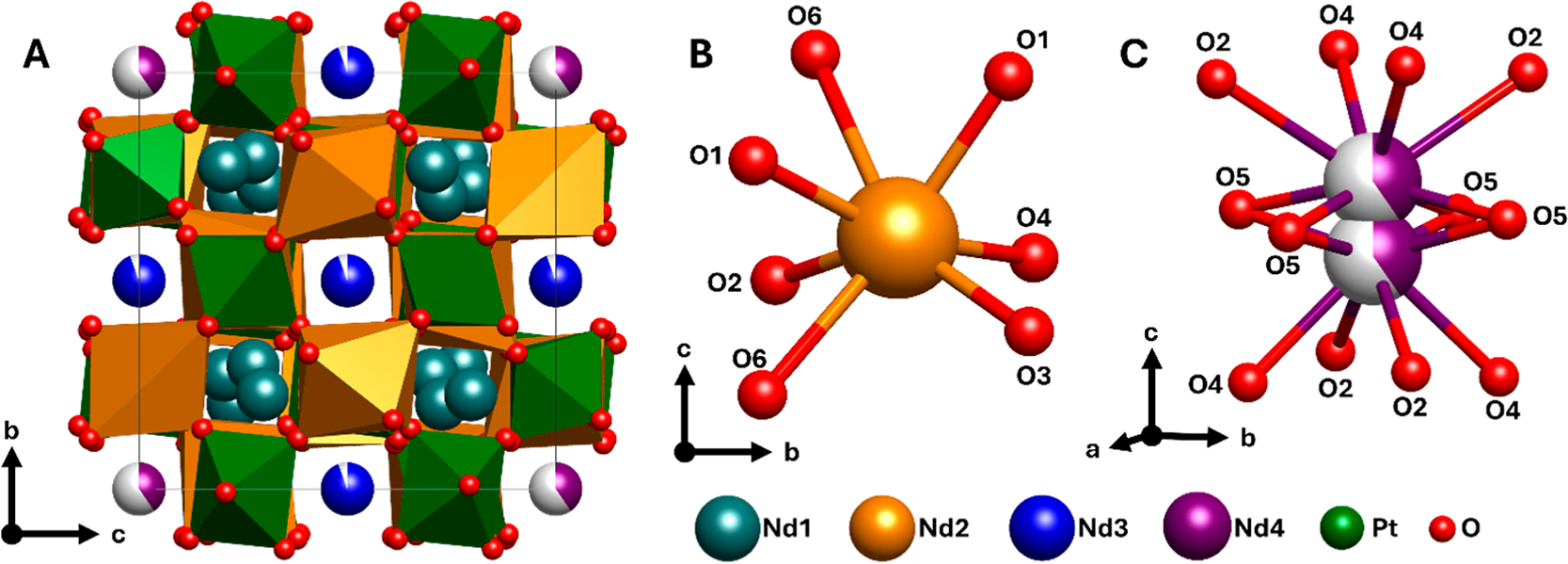
(**A)** Crystal structure of Nd_10.67_Pt_4_O_24_ with the neodymium sites illustrated in different colors. The oxygen coordination around the (**B)** Nd2 site and (**C)** Nd4 site. Partial occupancy as obtained from neutron powder diffraction data is illustrated with white area on the atoms Nd3 and Nd4.

The crystal structure of Nd_10.67_Pt_4_O_24_ is illustrated in Figure 2. The structure can be approximated as a Nd_2_(NdPt)O_6_ double perovskite where the Pt and Nd2 sites occupy corner-shared octahedra, Figure 2. However, a closer look into the crystal structure reveals that the Nd2 atom has coordination number (CN) = 7 and that the coordination polyhedron is actually edge-sharing with two of the PtO_6_ octahedra. The Nd2–O polyhedron is heavily distorted, with Nd2–O distances between d(Nd_2_–O_2_) = 2.300(2) Å and d(Nd_2_–O_6_) = 2.809(2) Å. For these reasons, the crystal structure has a complexity beyond that of the regular double perovskite type.^13^

Nd_10.67_Pt_4_O_24_ (see sections below for elaboration on stoichiometry) adopts a similar crystal structure as Ln_9_Sr_2_Ir_4_O_24_,^13^ including A_11_Re_4_O_24_ (A = Ca and Sr)^14,15^ and Ba_11_Os_4_O_24_.^16^ In these, the 5d cations (Re, Ir, Os, and Pt) take octahedral coordination and form along with some of the electropositive cations (Ln or alkaline earth) a complex double perovskite-like atomic arrangement. In several of these compounds, the 5d cations take mixed oxidations states, with charge ordering at two octahedral sites; their formal oxidation states are listed in the Supporting Information.

In Nd_10.67_Pt_4_O_24_, the Pt atoms take a quite regular octahedral environment, with an average Pt–O distance of 2.0203 and 2.0324 Å and distortion index () of 1.135 and 0.454% for Pt1 and Pt2, respectively. These bond lengths are fully consistent with expectations for Pt(IV). Based on Shannon radii,^30^ one notes that Nd–Pt–O would fulfill the pyrochlore stability criterion, however, barely the Goldschmidt *t*-factor criterion for the perovskite structure (here calculated *t* = 0.71). This structure type has been discussed in detail by Ferreira et al.,^13^ both in terms of a framework structure based on Nd1 and Nd2 polyhedra and in terms of unique chains of edge-sharing coordination polyhedra.

From the 3D ED collected by cRED, we obtained the refined composition Nd_10.733(16)_Pt_4_O_24_. In the model, both the Nd3 and Nd4 sites show partial occupancy, with refined occupancies from 3D ED of 0.988(6) and 0.379(5), respectively. Additionally, the Nd4 atom is displaced out of the center of the (deformed) cuboctahedron and obtains thereby an improved bonding situation. The split position reflects hence a double-well potential for the best occupation of the Nd4 cation within the large void. To shed more light on the nonstoichiometry and the split position behavior suggested by 3D ED and powder synchrotron X-ray diffraction (see Supporting Information), powder neutron diffraction data was collected. Specifically, the different contrast of neutrons compared to electrons and X-rays makes neutrons a better probe for oxygen positions, which provides a means to unambiguously verify the oxygen environment along with the neodymium nonstoichiometry (Table 1).

The structural model from 3D ED is in excellent agreement with the neutron data. By restricting the occupancy of Nd4 to unity while constraining all neodymium sites to have the same thermal displacement parameters, we observe some discrepancies in the fitted patterns and an *R*_wp_ of 3.26%. The discrepancies are reduced by turning to individual thermal displacement parameters and in particular when refining the Nd4 occupation number. It is thus clear that the Nd4 site displays cation vacancies. By restricting all the neodymium sites to have the same thermal displacement parameters and allowing refinement of the Nd4 occupancy, we reach a final *R*_wp_ of 2.09%. The occupancy of Nd4 is 0.404(3). This corresponds to a final composition of Nd_10.808(6)_Pt_4_O_24_, only slightly above the values obtained from 3D ED (Nd_10.733(16)_Pt_4_O_24_) and synchrotron diffraction (Nd_10.796_Pt_4_O_24_).

**Figure 3:**
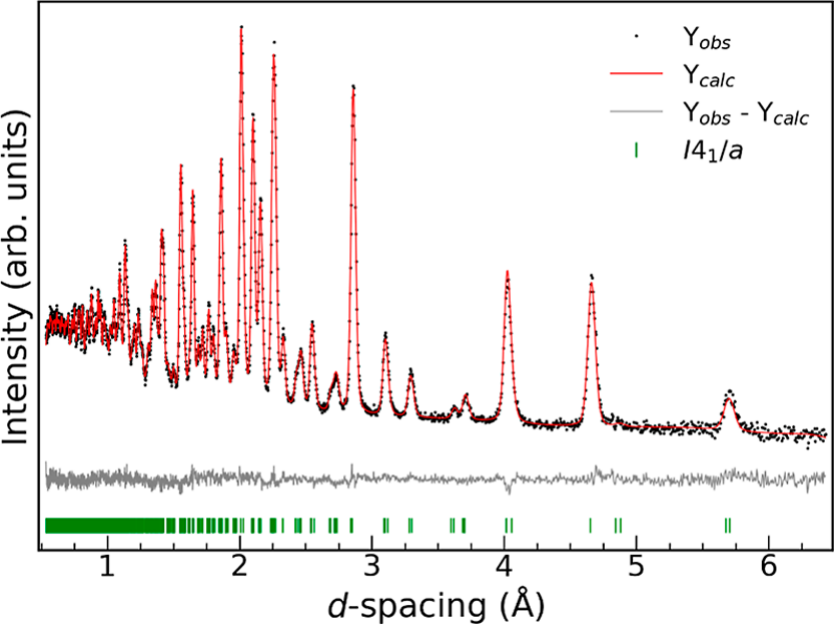
Rietveld refinement of neutron powder diffraction data from the third detector bank at GEM. The diagram shows the measured data (black dots), the calculated plot (red line), and the difference curve (gray). The Bragg reflection positions are given by green ticks.

We further evaluated the option of vacancies at other Nd sites and find that the Nd3 site occupancy consistently converges to 0.947(4), with the *R*_wp_ decreasing to 2.04%. The final refinements thus have an occupancy of 0.947(4) and 0.401(3) on the Nd3 and Nd4 sites, respectively. This yields an overall composition of Nd_10.696(1)_Pt_4_O_24_, which is in excellent agreement with values obtained from 3D ED and synchrotron diffraction. The composition is also close to what is expected from charge neutrality, namely, Nd_10.67_Pt_4_O_24_. Assuming full Pt and O occupancy, a fixed oxidation state + IV for Pt, and a valence state of + III for Nd, we obtain that the neodymium occupancy must be –(24 × (–2) + 4 × 4)/3 = 10.67. Owing to charge neutrality, we observe cation vacancies at the Nd3 and Nd4 sites, and the composition shows a clearly A-site cation-deficient compound. Charge neutrality is thus obtained by Nd vacancies and not mixed valence on Pt. This implies that Nd_10.67_Pt_4_O_24_ can be described as Nd_11–δ_Pt_4_O_24_ with δ = 0.33. The final structural model from neutron powder diffraction is given in Table 3, and the refinement of the third detector bank is shown in Figure 3. Refinements of the other detector banks and bond lengths are given in Supporting Information Figures S3–S7.

**Table 3:** Atomic Coordinates and Isotropic Displacement Parameters for Nd_10.696(1)_Pt_4_O_24_ Based on Refinement of Neutron Powder Diffraction Data^a^

atom	*x*	*y*	*z*	Biso (Å^2^)	Occ	Wyckoff
Nd1	0.72624(12)	0.70380(11)	0.50814(9)	0.741(14)	1	16f
Nd2	0.79456(10)	0.47697(12)	0.66240(7)	0.741(14)	1	16f
Nd3	0	0.5	0.48973(10)	0.741(14)	0.947(4)	8e
Nd4	0.5	0.5	0.5344(2)	0.741(14)	0.401(3)	8e
Pt1	0.75	0.5	0.375	0.293(19)	1	8c
Pt2	0	0.75	0.625	0.323(19)	1	8d
O1	0.90678(15)	0.32564(17)	0.71902(10)	0.969(13)	1	16f
O2	0.65140(16)	0.64422(17)	0.36876(12)	0.969(13)	1	16f
O3	0.89141(17)	0.61478(17)	0.59348(10)	0.969(13)	1	16f
O4	0.88048(18)	0.6205(2)	0.39771(10)	0.969(13)	1	16f
O5	0.5087(2)	0.70718(15)	0.50388(10)	0.969(13)	1	16f
O6	0.66503(16)	0.89043(18)	0.45295(12)	0.969(13)	1	16f

a Space group I4_1_*/a* with lattice parameters *a* = 11.3522(4) and *c* = 16.2122(5) Å. Calculated standard deviations in parentheses.

Solid solubility and site disorder have been reported for the iridate analogues, although there are mixed states of Ir(IV) and Ir(V) cations. In La_9_(Sr_0.925_La_0.075_)_2_Ir_4_O_24_, it was found that La and Sr are distributed over two of the 8e Ln sites, corresponding to the Nd3 and Nd4 sites in our case.^13^ For the current sample, there exists no solid solubility that could provide a similar site disorder. However, for both Nd_10.67_Pt_4_O_24_ and La_9_(Sr_0.925_La_0.075_)_2_Ir_4_O_24_, the Nd4 (and Nd3) site is just partly occupied, occupation numbers being, respectively, 0.33 and 0.50.^13^ This partial occupancy is required to ensure charge neutrality. One may therefore speculate whether a potential compound of Nd_10_SrPt_4_O_24_ may exist, without cation vacancies and split positions. For the compounds A_11_Re_4_O_24_ (A = Ca and Sr)^14,15^ and Ba_11_Os_4_O_24_^16^ with mixed valence states, Re(VI) and Re(VII), and likewise Os(VI) and Os(VII), there are no such A-site vacancies, Table 4. For the latter Ba_11_Os_4_O_24_,^16^ the relevant Ba cation is located to the 4a site at (0,1/4,1/8) which corresponds to the center between the split positions in Figure 2 where Nd4 takes a partly filled 8e site.

**Table 4:** Formal Oxidation State for the 5d Cations in Isostructural Compounds, in Which Charge Neutrality Is Obtained by Mixed Valence, whereas in Nd_10.67_Pt_4_O_24_ by Vacancies on Nd^a^

compound	specie I	specie II	ratio	charge balance	refs
A_11_Re_4_O_24_ (A = Ca and Sr)	Re(VI)	Re(VII)	1:1	11*2 + 2*6 + 2*7 – 24*2	(14,15)
A_11_Os_4_O_24_ (A = Sr and Ba)	Os(VI)	Os(VII)	1:1	11*2 + 2*6 + 2*7 – 24*2	(16)
La_9_Sr_2_Ir_4_O_24_	Ir(IV)	Ir(V)	3:1	9*3 + 2*2 + 3*4 + 1*5 – 24*2	(13)
Nd_10.67_Pt_4_O_24_	Pt(IV)			10.67*3 + 4*4 – 24*2	this work

a For the charge balance calculations, the charges of the 5d-element specie are given in bold.

The aspects of the correct positions for the Nd4 site were also evaluated based on the neutron powder diffraction refinements. If Nd4 were located at the 4a site (1/2, 1/2, 1/2), it would take a deformed cuboctahedral 12-fold coordination. However, the Nd–O bonds are in that case not favorable, four short 2.3549(18) Å and eight long 3.0736(19)–3.188(2) Å for Nd in the 4a site, compared to the refined bond lengths given in Table 5 for the 8e site. Hence, by shifting the Nd cation out of the center, the coordination changes and improves. Four of the 12 Nd–O bonds become significantly elongated, and a more favorable bonding situation can be achieved by shifting Nd4 in the vertical direction of the polyhedron, i.e., into the split position. In the Rietveld refinements of the neutron data, when moving Nd4 from (1/2, 1/2, z) with *z* ≈0.53 to (1/2, 1/2, 1/2), the *R*_wp_ increases from 2.04 to 2.4%. We therefore reject that Nd4 is on the 4a site and conclude that the 8e site is correct. We note that the O5 site is close to having coordinates (1/2, *y*, 1/2); however, (1/2, *y*, 1/2) is not a special position in I4_1_*/a*; it is still the 16f site.

**Table 5:** Obtained Nd4–O Interatomic Distances from Neutron Powder Diffraction Refinements

atoms	distance (Å)	distance (Å)
Nd4–O_2_	2.846(3) × 2	3.584(4) × 2
Nd4–O_4_	2.662(3) × 2	3.525(4) × 2
Nd4–O_5_	2.4053(19) × 2	2.434(2) × 2

## Thermal Stability and Evaluation of the Oxidation State of Platinum in Nd_10.67_Pt_4_O_24_

**Figure 4:**
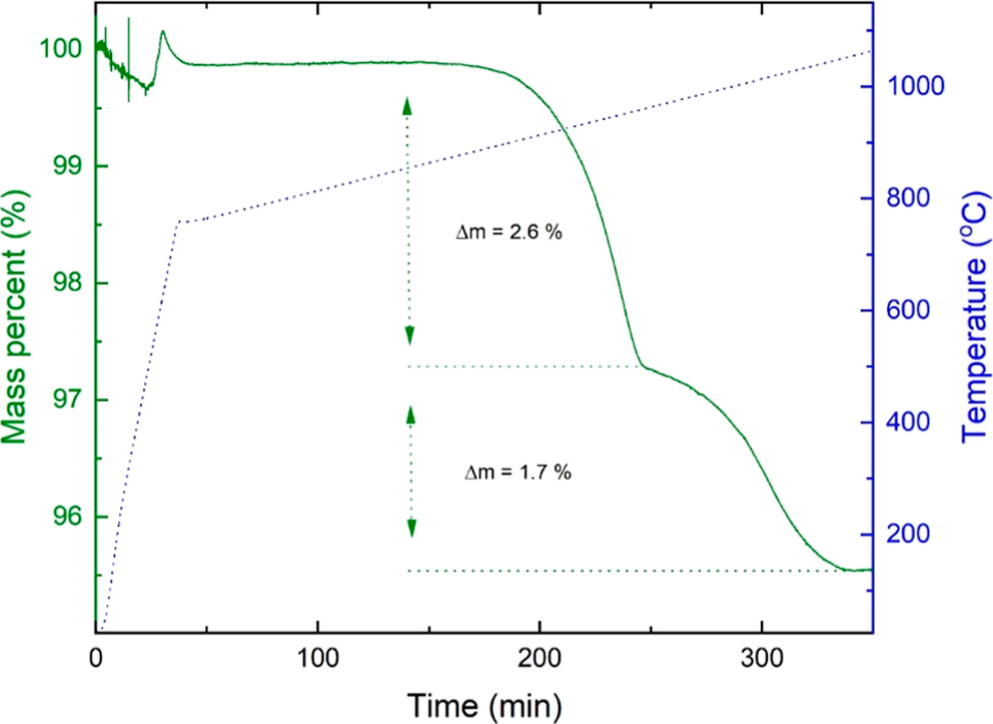
TGA of Nd_10.67_Pt_4_O_24_ upon heating at a rate of 20 °C/min between 25 and 750 °C, followed by a heating rate of 1 °C/min to 1100 °C. The gas atmosphere over the sample is 33 vol % O_2_ in N_2_.

To verify the oxidation state of platinum in Nd_10.67_Pt_4_O_24_, we evaluated the thermal stability and its decomposition products by TGA sending a 33 vol % O_2_ in N_2_ gas mixture over the sample. Upon heating at a rate of 20 °C/min between 25 and 750 °C, followed by a 1 °C/min ramping rate to 1100 °C, Nd_10.67_Pt_4_O_24_ undergoes three mass loss events, Figure 4. A small mass loss is observed below 400 °C, which probably reflects evaporation of adsorbed water or chemisorbed water (hydroxides) and/or CO_2_ (carbonate oxides). The second and third events have a total mass loss of 4.3%, corresponding well to complete reduction of Nd_10.67_Pt_4_O_24_ into Nd_2_O_3_, Pt metal, and O_2_, which has a theoretical mass loss of 4.4%. Similarly, a mass loss of 4.3% is observed for the thermal decomposition of Nd_10.67_Pt_4_O_24_ in N_2_ gas (not shown). The powder X-ray diffraction pattern of the TGA residue after decomposition at 1100 °C consists entirely of Nd_2_O_3_ and Pt; see Supporting Information Figure S8.

The TGA data using a slow heating rate of 1 °C/min above 750 °C (Figure 4) show that the onset temperature of the decomposition is at ∼930 °C. Additionally, it became possible to study a distinct plateau at around 960 °C, indicating the likely presence of an intermediate phase. To isolate this intermediate phase, an experiment was carried out within the TGA apparatus where the heating was terminated after reaching the plateau weight. The powder X-ray diffraction pattern for the TGA residue, after cooling, is shown in Supporting Information Figure S9. Sharp characteristic diffraction peaks of Pt are observed along with very broad yet distinct diffraction features of an ill-defined product. We suggest that the product represents a two-phase mixture between Pt and an unknown Nd-enriched platinate, probably a Pt(II) phase. Indeed, Rietveld refinements assuming the presence of an Nd_4_PtO_7_ phase (not earlier reported) that takes an La_4_PtO_7_ type structure^10^ are consistent with the observed powder X-ray diffraction data, see Figure S9.

The TGA experiments show no indications of oxygen nonstoichiometry below 900 °C. There is no indication of any presence of Pt(II) or a mixed valence state that could provide charge balance as an alternative to Nd vacancies. Note that no such vacancies occur in the isostructural rhenates and osmates (Table 4) due to a redox flexibility of the involved 5d cations.

## Conclusions

In summary, we have prepared a new Nd platinate, Nd_10.67_Pt_4_O_24_, and described its crystal structure, structural chemistry, and vacancies in detail by a combination of diffraction probes and thermogravimetry. The compound is structurally related to several complex rhenates, iridates, and osmates with a double perovskite-like atomic arrangement. In contrast to these compounds, charge neutrality in Nd_10.67_Pt_4_O_24_ is obtained by neodymium vacancies to maintain tetravalent platinum. Pt(IV) adopts an octahedral coordination in the structure. Nd_10.67_Pt_4_O_24_ is thermally stable up to some 900 °C in a 33 vol % O_2_ in N_2_ gas atmosphere and is readily obtained according to a soft chemistry synthesis approach or by a direct reaction using gaseous PtO_2_. The latter is relevant during the Ostwald process for nitric acid/fertilizer production when a Nd_2_O_3_-based catchment system is used.

## Supporting Information Available

Additional information about 3D ED refinements and rocking curve profiles, atomic coordinates and Rietveld refinement from X-ray diffraction data, Rietveld refinement and bond lengths from neutron diffraction data, and Rietveld refinement of the decomposition product and intermediate phase from TGA (PDF)
Crystallographic data of Nd_10.67_Pt_4_O_24_ (CIF)

## Supplementary Material

ao5c00031_si_001.pdf

ao5c00031_si_002.cif
