# Supplementary material for: Crystal Structure of Nd10.67Pt4O24, a New Neodymium Platinate
Source: ACS Omega. 2025 Mar 19;10(12):12487–94. doi: 10.1021/acsomega.5c00031 (PMC11966264; doi:10.1021/acsomega.5c00031)
Supplement: Supplementary file 1 — ao5c00031_si_001.pdf [file ao5c00031_si_001.pdf]

Supplementary information: Crystal structure of $Nd_{10.67}Pt_4O_{24}$, a new neodymium platinate

Øystein Slagtern Fjellvåg[†], Helmer Fjellvåg[‡,], Julie Hessevik[‡], Anja Olafsen Sjåstad[‡] and Gwladys Steciuk[§]*

[†] Department for Hydrogen Technology, Institute for Energy Technology, P.O. Box 40, Kjeller NO-2027, Norway;

[‡] Center for Materials Science and Nanotechnology, Department of Chemistry, University of Oslo, N-0315 Oslo, Norway,

[§] Institute of Physics of the CAS, Na Slovance 1999/2, 182221 Prague 8, Czech Republic

TABLES

- Atomic coordinates from synchrotron X-ray diffraction are given in Table S1.
- Table S2 shows atomic distances from powder neutron diffraction refinements.

FIGURES

- The Rocking curve profiles from the 3D ED refinements are given in Figure S1.
- Figure S2 shows the Rietveld refinement of the synchrotron X-ray diffraction data.
- Figure S3-S7 show the refinements of the different detector banks of the powder neutron diffraction data.
- Figure S8 shows the Rietveld refinement of the decomposition product from TGA, which is a mix between Pt and Nd_2O_3.
- Figure S9 shows the Rietveld refinement of the intermediate phase refined with Pt and La_4PtO_7 structures.

The single crystal structure obtained by 3D ED was evaluated at the powder scale by means of Rietveld refinements of the synchrotron powder diffraction data (Table 1 main text). An excellent agreement between the model and the experimental data validated the structural model. A composition of $Nd_{10.796}Pt_4O_{24}$ was obtained from the refinements. The refinement details are given in Table 1, the obtained atomic coordinates are given in the Table S1, and the refinement is shown in Figure S2.

Table S1. *Atomic coordinates and isotropic displacement parameters for $Nd_{10.67}Pt_4O_{24}$ based on refinement of synchrotron powder diffraction data. Space group $I4_1/a$ with lattice parameters a = 11.35203(11) Å and c = 16.21114(18) Å. Calculated standard deviations in parentheses. The refined occupation number for the Nd4 site is 0.398(5).*

**Atom**	**x**	**y**	**z**	**Uiso (Å^2)**	**Wyckoff**
Nd1	0.72578(18)	0.70409(15)	0.50945(14)	0.0113(5)	*16f*
Nd2	0.79315(14)	0.4759(2)	0.66280(9)	0.0160(5)	*16f*
Nd3	0	0.5	0.49110(15)	0.0194(6)	*8e*
Nd4	0.5	0.5	0.5341(3)	0.010(2)	*8e*
Pt1	0.75	0.5	0.375	0.0067(5)	*8c*
Pt2	0	0.75	0.625	0.0059(4)	*8d*
O1	0.9105(15)	0.3304(19)	0.7148(9)	0.0052(17)	*16f*
O2	0.6473(15)	0.6366(18)	0.3723(12)	0.0052(17)	*16f*
O3	0.8877(15)	0.6033(17)	0.5903(10)	0.0052(17)	*16f*
O4	0.8719(17)	0.632(2)	0.4015(11)	0.0052(17)	*16f*
O5	0.502(2)	0.7043(13)	0.5028(7)	0.0052(17)	*16f*
O6	0.6761(18)	0.8880(17)	0.4556(12)	0.0052(17)	*16f*

Table S2: Metal-oxygen bond distances from Rietveld refinements of neutron powder diffraction data at room temperature.

Bond	Distance (Å)	Distance (Å)
Nd1-O1	2.500(2)	
Nd1-O2	2.507(2)	2.605(2)
Nd1-O3	2.540(2)	
Nd1-O4	2.677(2)	2.734(2)
Nd1-O5	2.430(3)	2.471(3)
Nd1-O6	2.403(2)	
Nd2-O1	2.327(2)	2.358(2)
Nd2-O2	2.300(2)	
Nd2-O3	2.215(2)	
Nd2-O4	2.565(2)	
Nd2-O5	2.877(2)	
Nd2-O6	2.698(2)	2.809(2)
Nd3-O2	2.954(2) × 2	
Nd3-O3	2.459(2) × 2	
Nd3-O4	2.437(2) × 2	
Nd3-O6	2.433(2) × 2	
Nd4-O1		
Nd4-O2	2.846(3) × 2	3.584(4) × 2
Nd4-O3		
Nd4-O4	2.662(3) × 2	3.525(4) × 2
Nd4-O5	2.4053(19) × 4	2.434(2) × 4
Nd4-O6		
Pt1-O2	1.9859(19) × 2	
Pt1-O4	2.049(2) × 2	

Pt1-O5	2.0254(16) × 2
Pt1-O1	2.0446(17) × 2
Pt1-O3	2.0340(19) × 2
Pt1-O6	2.019(2) × 2

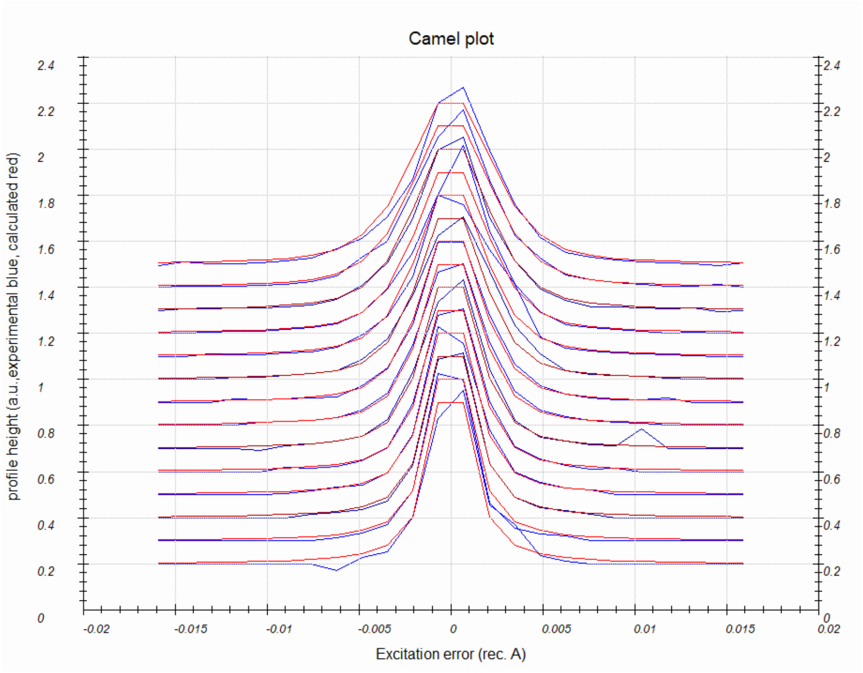

Figure S1: Plot of the rocking-curve profiles (Camel plot) of the experimental 3D ED data. The lowest blue curve is the average observed rocking curve in the range of 0.2 to 0.3 Å−1, and the next ones are obtained by steps of 0.1 Å−1. The red dotted curves are calculated from the *Rocking curve width* = 0.00105 $Å^{-1}$, the apparent mosaicity = 0.04796 °, and the tilt semi-angle = 0.2 °. Reflections are involved in the Camel plot for I > 5*σ(I) [31].

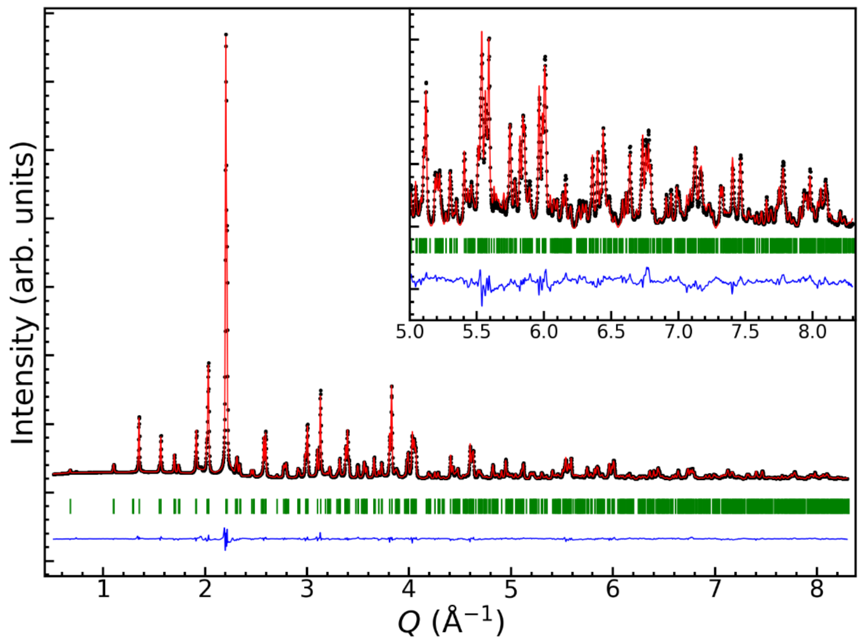

Figure S2. Rietveld refinement of $Nd_{10.67}Pt_4O_{24}$ from synchrotron powder diffraction data with a wavelength 0.25509 Å. The diagram shows the measured data (black dots), the calculated plot (red line), and the difference curve (blue). The reflection positions are given by green ticks. Insert show the fit to high-angle data.

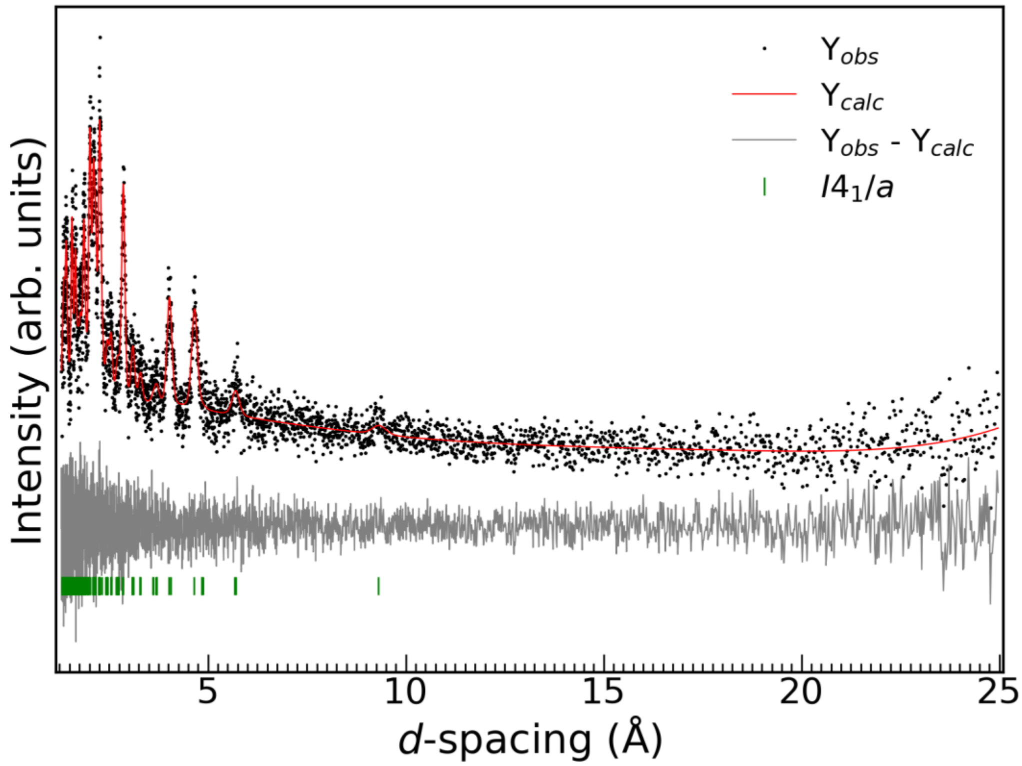

Figure S3: Rietveld refinement of neutron powder diffraction data from the first detector bank at GEM.

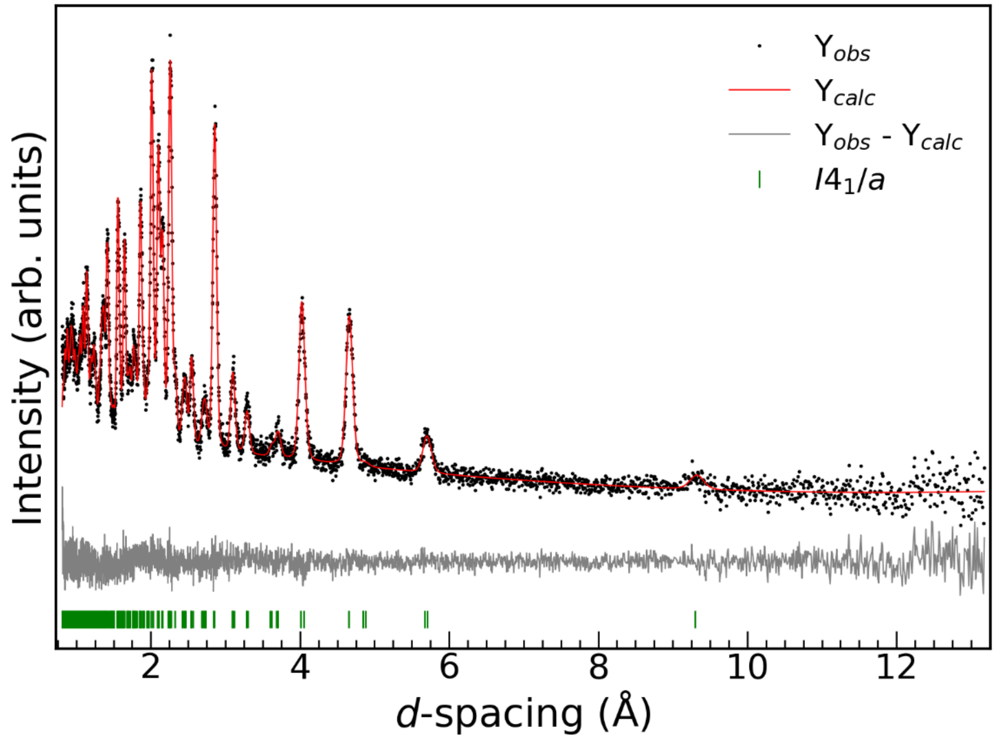

Figure S4: Rietveld refinement of neutron powder diffraction data from the second detector bank at GEM.

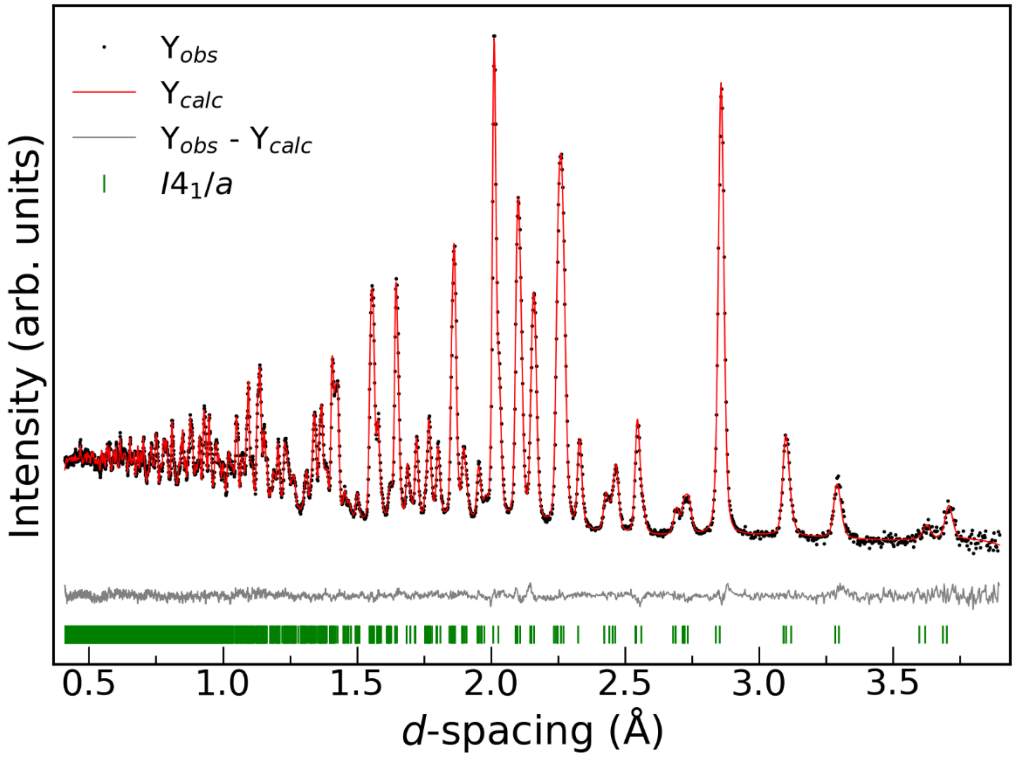

Figure S5: Rietveld refinement of neutron powder diffraction data from the fourth detector bank at GEM.

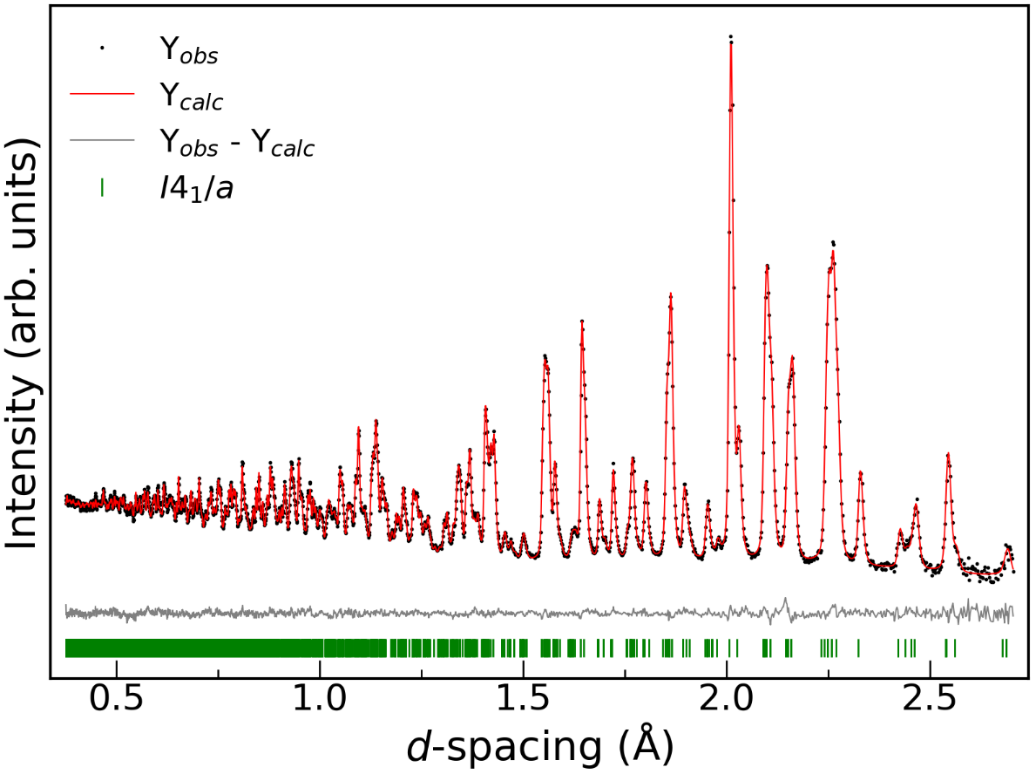

Figure S6: Rietveld refinement of neutron powder diffraction data from the fifth detector bank at GEM.

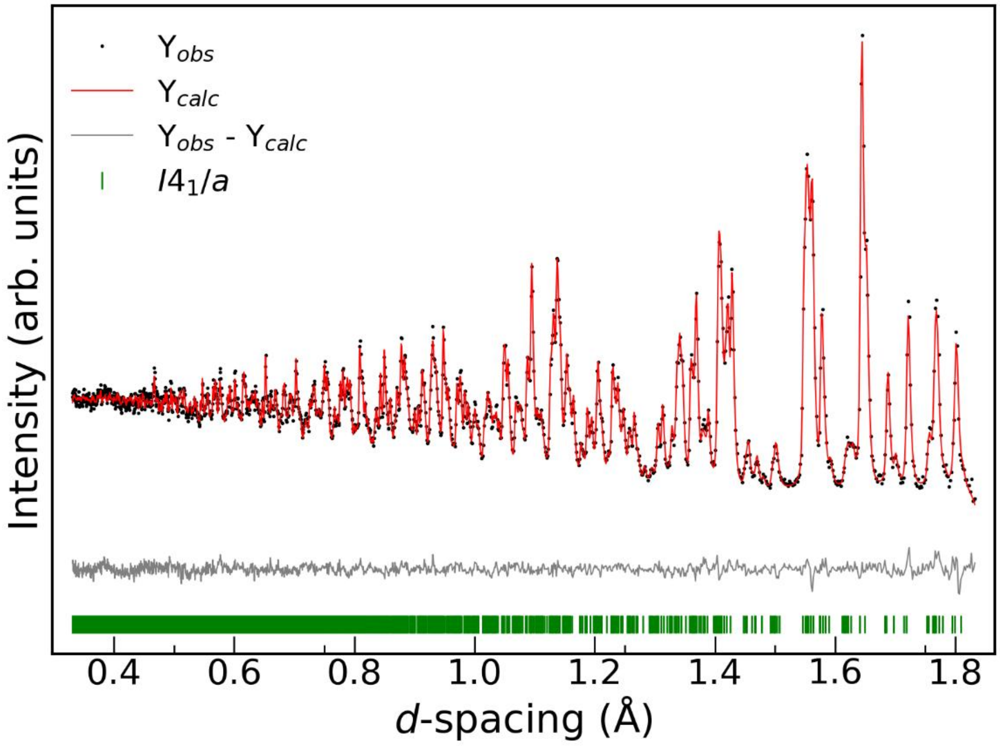

Figure S7: Rietveld refinement of neutron powder diffraction data from the sixth detector bank at GEM.

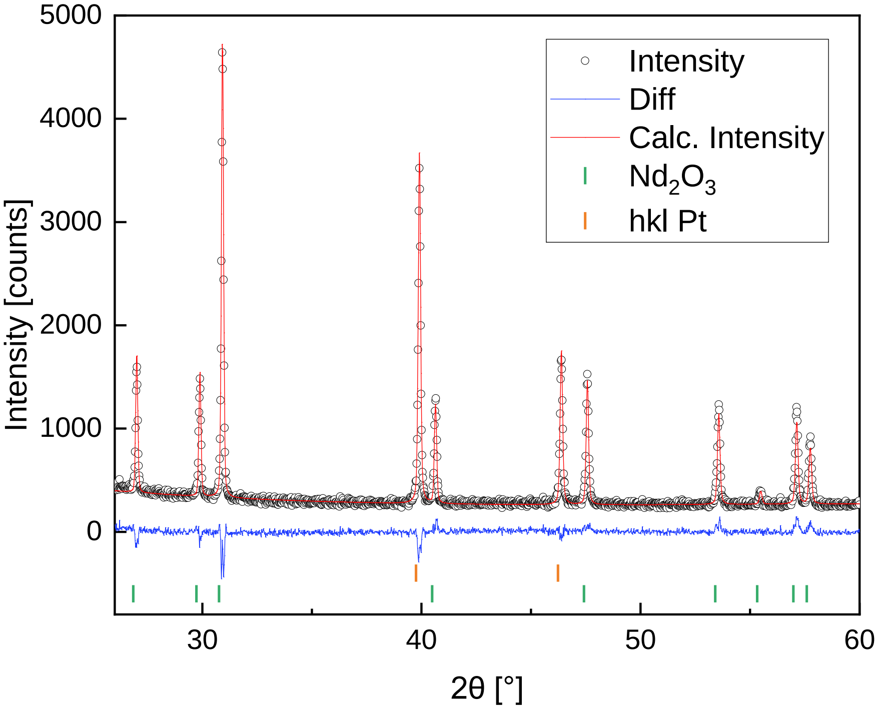

Figure S8. Rietveld refinement of powder X-ray diffraction data after thermogravimetric analysis of $Nd_{10.67}Pt_4O_{24}$ in a N_2 gas atmosphere to 1100 °C. The refinement shows a mixture of Nd_2O_3 and Pt.

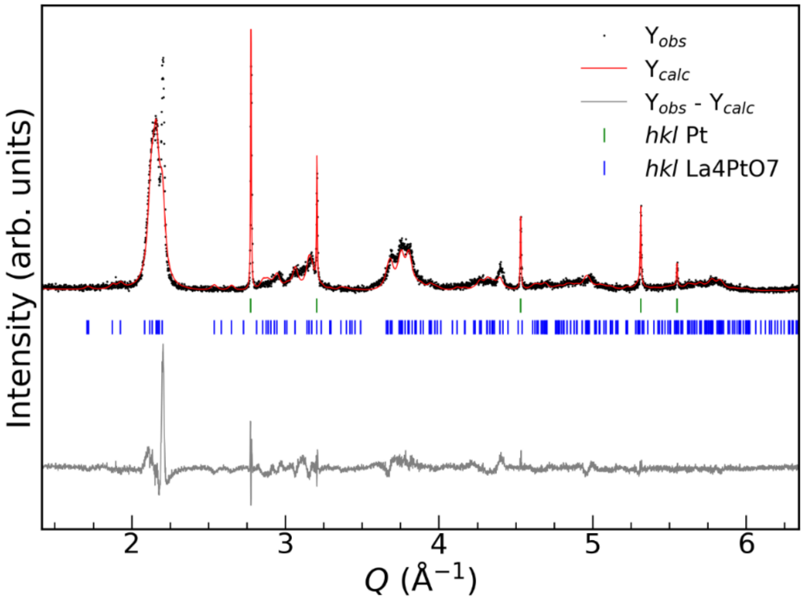

Figure S9. Rietveld refinement using Pt and the La_4PtO_7 phase obtained by decomposition of $Nd_{10.67}Pt_4O_{24}$ in a 33 vol. % O_2 in N_2 gas atmosphere up to 960 °C. No atomic coordinates were refined, values from Ref [1] was used. Background, sample displacement, lattice parameters and peak

broadening were refined. Refined lattice parameters: Pt: a = 3.9227(2) Å, La_4PtO_7: a = 9.493(2) Å, b = 3.9857(7) Å, c = 9.223(2) Å, β = 91.55(2) Å.

References:

[1] T.J. Hansen, R.B. Macquart, M.D. Smith, H.-C. zur Loye, Crystal growth and structures of three new platinates: Ln_3NaPtO_7 (Ln = La, Nd) and $La4PtO_7$, Solid State Sci. 9 (2007) 785–791. https://doi.org/10.1016/j.solidstatesciences.2007.06.014.
